# Mitogenome Characterization of Four *Conus* Species and Comparative Analysis

**DOI:** 10.3390/ijms24119411

**Published:** 2023-05-28

**Authors:** Hao Wang, Xiaopeng Zhu, Yuepeng Liu, Sulan Luo, Dongting Zhangsun

**Affiliations:** 1 Key Laboratory of Tropical Biological Resources of Ministry of Education, Hainan University, Haikou 570228, China; wanghao230501@126.com (H.W.); liuyuepeng2018@outlook.com (Y.L.); 2 School of Medicine, Guangxi University, Nanning 530004, China; biozxp@163.com

**Keywords:** cone snail, mitogenomes, phylogenetic analysis, PCG, COX1

## Abstract

Cone snails, as a type of marine organism, have rich species diversity. Traditionally, classifications of cone snails were based mostly on radula, shell, and anatomical characters. Because of these phenotypic features’ high population variability and propensity for local adaptation and convergence, identifying species can be difficult and occasionally inaccurate. In addition, mitochondrial genomes contain high phylogenetic information, so complete mitogenomes have been increasingly employed for inferring molecular phylogeny. To enrich the mitogenomic database of cone snails (Caenogastropoda: Conidae), mitogenomes of four *Conus* species, i.e., *C. imperialis* (15,505 bp), *C. literatus* (15,569 bp), *C. virgo* (15,594 bp), and *C. marmoreus* (15,579 bp), were characterized and compared. All 4 of these mitogenomes included 13 protein-coding genes, 2 ribosomal RNA genes, 22 tRNA genes, and non-coding regions. All the Protein Codon Genes (PCGs) of both newly sequenced mitogenomes used TAA or TAG as a terminal codon. Most PCGs used conventional start codon ATG, but an alternative initiation codon GTG was detected in a gene (NADH dehydrogenase subunit 4 (nad4)) of *C. imperialis*. In addition, the phylogenetic relationships were reconstructed among 20 *Conus* species on the basis of PCGs, COX1, and the complete mitogenome using both Bayesian Inference (BI) and Maximum Likelihood (ML). The phylogenetic results supported that *C. litteratus*, *C. quercinus*, and *C. virgo* were clustered together as a sister group (PP = 1, BS = 99), but they did not support the phylogenetic relation of *C. imperialis* and *C. tribblei* (PP = 0.79, BS = 50). In addition, our study established that PCGs and complete mitogenome are the two useful markers for phylogenetic inference of *Conus* species. These results enriched the data of the cone snail’s mitochondrion in the South China Sea and provided a reliable basis for the interpretation of the phylogenetic relationship of the cone snail based on the mitochondrial genome.

## 1. Introduction

Cone snails belong to the phylum Mollusca, Gastropod, Conidae, and they are well known for their conotoxins. Like most Neogastropoda species, cone snails are dioecious and fertilized in vivo. Adult males and females mate and fertilize to produce a collection of spawns that develop in an oocyst, and then the spawns develop into free-swimming disc larvae, which then develop into adults [1]. Most cone snails feed on polychaete worms, and others are either molluscivorous or piscivorous, with few species having more than one feeding pattern, which can generate an estimated 100,000 distinct conotoxins [2,3,4]. Additionally, owing to their exquisite potency and selective inhibitors of ion channel in the human brain, conotoxins have potential applications in medicine and physiology [5,6,7]. To comprehend how the great species diversity of the cone snails was generated and to interpret the origin of the different diet specializations, reconstructing a statistically robust phylogeny of cone snails is needed, which can enhance and improve the current discovery of pharmacologically important conotoxins [8,9].

Classifications of cone snails in the previous century were based mostly on the shell, radula, and anatomical characters [10,11]. These traditional methods of identifying species can be difficult and occasionally inaccurate because of the phenotypic features’ high population variability and propensity for local adaptation and convergence. With the development of molecular biology, classification methods using specific molecular markers are increasingly favored by researchers. Single mitochondrial molecular markers, such as 16srRNA and COX1 (cytochrome c oxidase subunit 1), have been widely used in the phylogenesis of metazoan [8,12] However, phylogenetic information provided by a single gene is limited. Compared with a single gene, mitochondrial genomes are more informative. In addition, it is necessary to assess other independent markers to reconstruct the phylogeny of *Conus.*

The complete mitochondrial genome of most metazoans are circular or double-stranded molecules, ranging in size from approximately 14 to 18 kb. Due to its maternal inheritance, stable structure, relatively high evolutionary rate, and lack of genetic recombination, molecular phylogenetic analysis has increasingly utilized complete mitogenomes; this helps to solve the problem faced by traditional molecular phylogenetic analysis that relies on a single or few selective markers [8,13,14,15,16]. Samuel Abalde et al. [17] found, by using PCGs and rRNA of the mitochondrial genome to construct phylogenetic trees, that the taxon *Lautoconus reticulatus*, which was traditionally considered to be a junior synonym of *Conus mercator*, is a valid species distinct from *L. mercator*. The mitochondrial genome is helpful in enhancing our comprehension of phylogenetics, species identification, population genetics, and molecular evolution in some taxa of *Conus*.

To date, more than 900 *Conus* species have been described and named [18], but the complete mitochondrial genomes of only 16 *Conus* species are included in the National Center for Biotechnology Information (NCBI) (Table S4). Currently, no prior research is reported on the mitochondrion of *C. imperialis*, *C. litteratus*, *C. virgo*, or *C. marmoreus* in the South China Sea. In this study, we aimed to enrich the mitogenomic database of the species of *Conus* and to analyze primarily the phylogenetic relationship of the species of *Conus* on the basis of the mitogenomes. We sequenced, annotated, and comparatively analyzed the complete mitogenomes of four species of *Conus* (i.e., *C. imperialis*, *C. litteratus*, *C. virgo*, and *C. marmoreus*). The features of their mitogenomes and a comparative analysis, including previous sequenced *Conus* mitochondrial genomes, are addressed. Moreover, we included another 16 species known from *Conus* for the mitogenomes to reconstruct their phylogenetic relationship among the 20 species with 3 different datasets. Overall, our results provide a basis for further phylogenetic analysis of the 20 species of *Conus*, and we analyze 3 markers (COX1, PCGs, and the complete mitogenome) of mitochondrion to make phylogenetic inferences of the *Conus* species.

## 2. Results

### 2.1. Genome Structure and Organization

**Figure 1:**
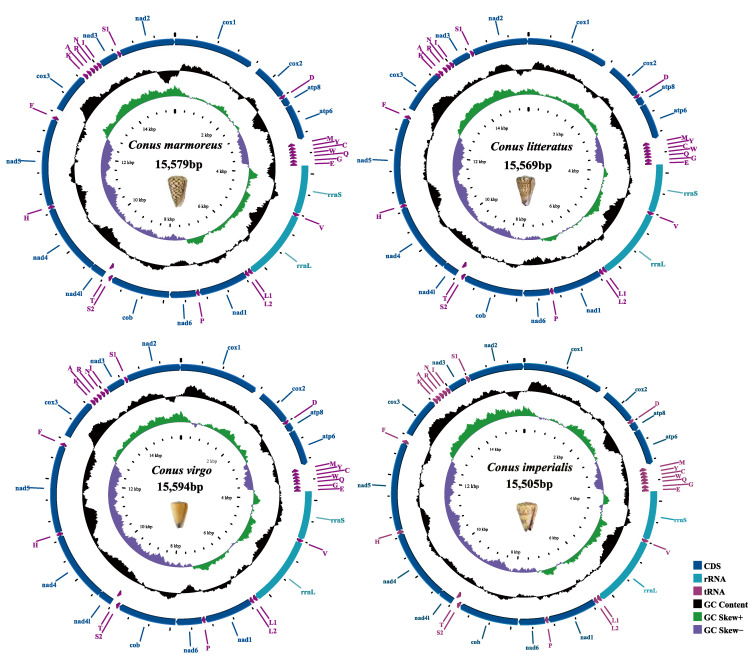
Circular maps of the four newly sequenced mitogenomes. A ventral picture of the shell is provided. The first and second circles of the map are CDS, rRNAs, and tRNAs on the positive and negative strands. The third circle indicates GC content, and the inwards part indicates that its GC content is lower than the genome-wide average GC content, with higher peaks indicating higher GC content. The fourth circle represents the GC-skew value.

The newly sequenced complete mitogenomes of *C. imperialis* (GeneBank accession number:), *C. litteratus* (GeneBank accession number:), *C. virgo* (GeneBank accession number:), and *C. marmoreus* (GeneBank accession number:) were 15,505 bp, 15,569 bp, 15,594 bp, and 15,579 bp, respectively. These 4 mitogenomes contained the same 37 genes (22 tRNAs, 13 PCGs (COX1-3 (cytochrome c oxidase genes), ND1-6 (NADH dehydrogenase genes), ND4L (NADH dehydrogenase gene), CYTB (cytochrome b), ATP6 (ATP synthase gene), and ATP8 (ATP synthase gene)), 2 rRNAs (12S and 16S), and a control region). These four newly sequenced mitogenomes had the same gene arrangement and orientation as the prior *Conus* mitochondrial genomes. Most genes (29 genes) of the mitochondrial genome were encoded on the majority strand (J-strand), and other genes (8 tRNAs) of the mitochondrial genome were encoded on the minority strand (N-strand) (Figure 1). The results showed that the locations of PCGs and RNA genes were conservative.

**Figure 2:**
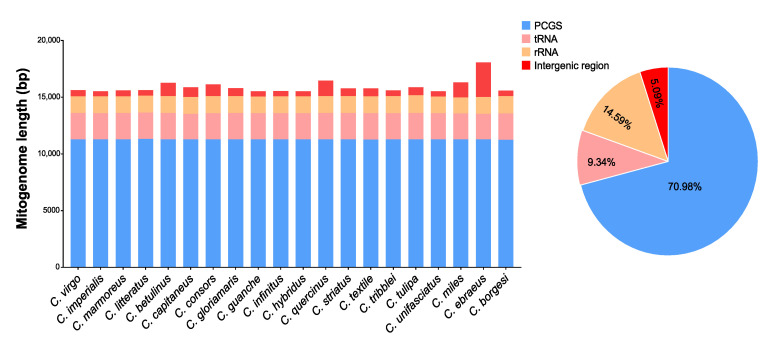
Length distribution of PCGs, tRNA, rRNA, and intergenic region in the mitogenomes of 20 species of *Conus*.

The lengths of the complete mitogenome among 20 cone snails (4 species of *Conus* in this study and the other 16 previous identified species of *Conus*) ranged from 15,505 bp to 18,031 bp, and the majority of the mitogenome lengths among them were conservative. In comparison, gene counts and lengths of PCGs, rRNA, and tRNA were conserved among the 20 species of *Conus* with various total mitogenome lengths, and among them, they occupied a large proportion in the mitogenome. The lengths of intergenic regions varied among the 20 species of *Conus* (Figure 2).

The noncoding regions of the mitogenome were composed of a control region (CR) and the intergenic spacers (IGSs). A single large non-coding region is commonly found in metazoan mitogenomes, containing signals for the initiation of transcription and replication [19,20]. The CR of our four newly sequenced mitogenomes were located at the conserved position between tRNA^Phe^ and cox3 with the length of 120 bp in *C. imperialis*, 128 bp in *C. litteratus*, 140 bp in *C. virgo*, and 140 bp in *C. marmoreus* (Table 1). These regions had a high level of length variation (Figure 2).

#### Nucleotide Composition

We compared the nucleotide composition of mitochondrial genomes of 4 cone snails with 16 previously identified species. The skew metrics of the mitochondrial genome of all available *Conus* showed negative AT-skew (−0.2 to −0.12) and positive GC-skew (0.06 to 0.19) (Table 1). The entire AT content of the complete mitochondrion was 66.05% in *C. litteratus*, 65.96% in *C. imperialis*, 64.42% in *C. virgo*, and 66.81% in *C. marmoreus*, which were similar to other *Conus* (Table 1). The majority of metazoan mitochondrion genomes shared a common trait that a higher representation of A+T content led to a subsequent bias in the corresponding encoded amino acids [21].

### 2.2. Protein-Coding Genes Analysis

#### Codon Preference Statistics

All *C. litteratus*, *C. imperialis*, *C. marmoreus*, and *C. virgo* contained 13 protein-coding genes, which were all coded on the majority strand (J-strand). In all newly sequenced mitogenomes, the usage patterns of the initial and terminal codon of *C. litteratus*, *C. imperialis*, *C. marmoreus*, and *C. virgo* were highly conservative. Their initial and terminal codons for the PCGs were compared with another sixteen species of *Conus*. Initiation codons of PCGs of all twenty species analyzed were the typical codon ATG. Nonetheless, there were some exceptions, i.e., the nad4 of *C. betulinus*, *C. capitaneus*, *C. striatus*, and *C. imperialis* started with GTG, the nad4 of *C. borgesi* started with ATA. As for termination codons, most PCGs utilized TAA or TAG as the termination codon, but a truncated stop codon T was detected in COX2 of *C. betulinus*, and TA was detected in nad4 of *C. betulinus*, *C. Guanche*, *C. infinitus*, and *C. unifasciatus* (Tables S1 and S2). The incomplete terminal codons were anticipated to be completed as TAA by post-transcriptional polyadenylation [22].

**Figure 3:**
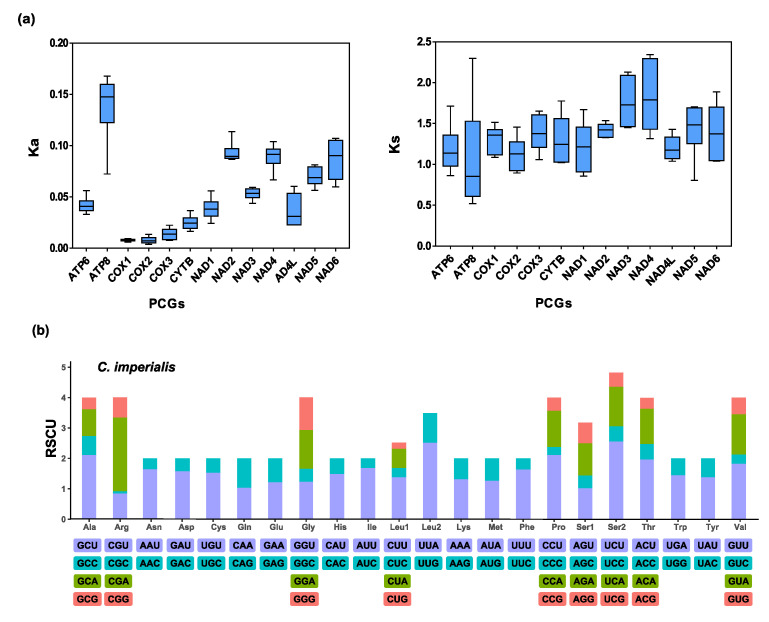
(**a**) Nucleotide substitution rates of conserved PCGs in mitogenomes of the four *Conus* species isolates. The order of conserved PCGs below the X-axis is in accordance with their arrangement on the mitogenome. (**b**) The relative synonymous codon usage (RSCU) of the mitogenomes, using *C. imperialis* as a model; others are shown in Figure S1. The order of amino acids below the X-axis and the color of codons correspond to their order of the letters.

The relative synonymous codon usage (RSCU) values of the four newly sequenced mitogenomes of *Conus* species are summarized (Figure 3 and Figure S1). All stop codons were removed from the calculation to prevent bias brought on by stop codons that were not complete. Codons rich in A or T were commonly used in the mitogenomes of *C. litteratus*, *C. imperialis*, *C. marmoreus*, and *C. virgo*, such as UUA (Leucine), GUU (Valine), UCU (Serine2), ACU (Threonine), and UAU (Tyrosine). On the contrary, the rarely used codons were from G+C-rich codon families, such as CCC (Proline) and CGC (Argnine). This indicated that the relative synonymous codon usage (RSCU) values were positively correlated with the content of A and T. All PCGs among these four newly sequenced *Conus* mitogenomes had a Ka value of >0 and Ks > 0, indicating that all these genes had synonymous substitution and nonsynonymous substitution (Figure 3). In addition, all PCGs in these four newly sequenced mitogenomes had Ka/Ks values of <1, indicating purifying selection for these genes.

### 2.3. Transfer RNA Genes

**Figure 4:**
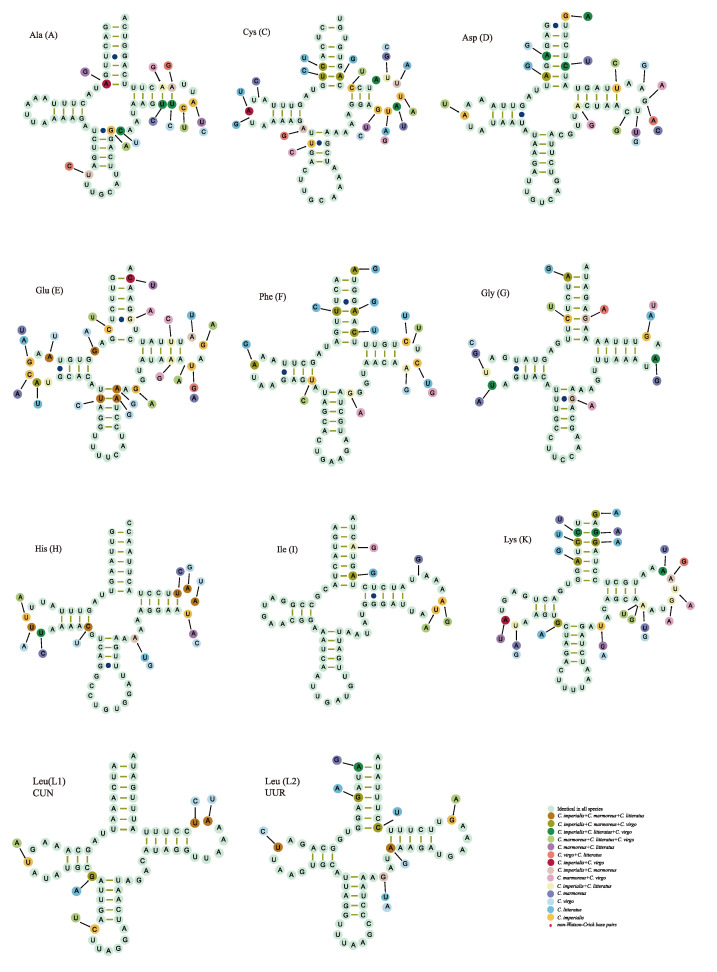

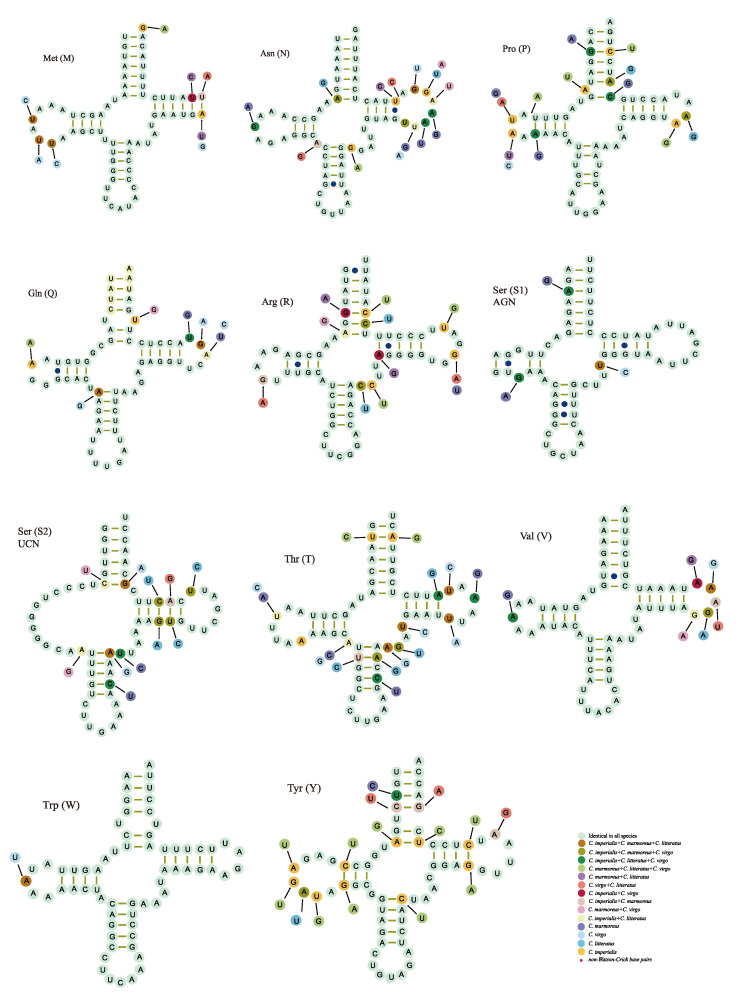
The putative tRNA second structure of four *Conus* species. The different colors refer to the nucleotide identity using the putative tRNA second structure of *C. imperialis* as a reference.

The mitogenomes of *C. litteratus*, *C. imperialis*, *C. marmoreus*, and *C. virgo* contained twenty-two typical tRNAs: two copies of trnS and trnL and one for the other amino acids. Most of the tRNAs in our analysis could be folded into a typical clover-leaf secondary structure, except for tRNAser(S2), which lacked the DHU arm (Figure 4). The length of tRNAs of our analyzed mitogenomes ranged from 65 to 71 bp of *C. litteratus*, 65–70 bp of *C. imperialis*, 58–71 bp of *C. marmoreus*, and 58–71 bp of *C. virgo*.

The comparative analysis of tRNA secondary structures showed that the percentage of identical nucleotides in the conserved tRNA was high, such as for tRNAHis(H), tRNAIle(I), tRNALeu(L1), and tRNAser(S1), and some other tRNAs (tRNACys(C), tRNAArg(R), and tRNATyr(Y)) had high levels of nucleotide variation.

### 2.4. Phylogenetic Analysis

**Figure 5:**
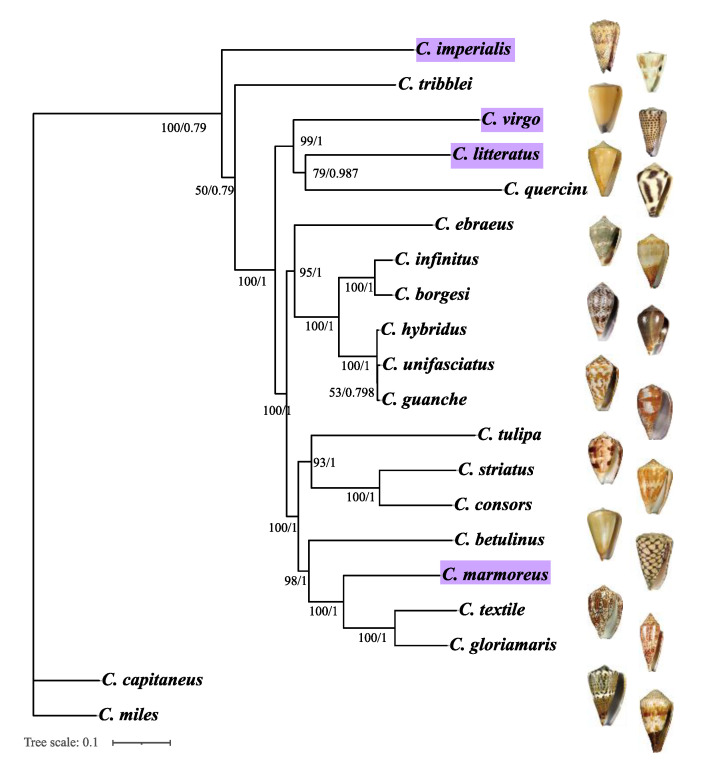
The phylogenetic tree from ML and BI analyses of 20 *Conus* species, using the phylogenetic tree from ML and BI based on complete mitogenome as a reference. The others are shown in Figure S2. The illustration of the shell of *C. litteratus*, *C. imperialis*, *C. marmoreus*, and *C. virgo* was photographed by our lab, while others refer to the database (https://www.marinespecies.org/ (accessed on 7 May 2023)). Tree topologies constructed by the three methods are in almost complete agreement. ML bootstrap value (BV) and BI posterior probability (PP) are separated by a slash on the node. The reconstructed ML/BI phylogram, using *C. capitaneus* and *C. miles* as outgroup. The purple refers to the species whose mitogenomes were newly sequenced in this work.

ISS value and ISS.c value are two indexes of substitution saturation tests; if the ISS value < ISS.c value and *p* < 0.05, this indicates that the dataset is not saturated and is suitable for phylogenetic analysis. The results of DAMBE analysis showed that three datasets (COX1, PCGs, and the complete mitogenome) had lower ISS values than the ISS.c (*p* ≤ 0.0001), which confirmed the suitability for phylogenetic analyses of these three datasets (Table S3). The partition-specific models and the best-fit partitioning scheme were used to construct the phylogenetic analysis (Table S6). The topological structures of all phylogenetic analyses based on Bayesian (BI) and Maximum Likelihood (ML) analyses were almost in agreement (Figure 5 and Figure S2). In contrast to nodes based on PCGs and the complete mitogenome, nodes in the phylogenetic tree based on COX1 had low support values. Among the phylogenetic trees constructed from the three datasets, the topology structure of the phylogenetic tree based on PCGs and the complete mitogenome was more stable than that based on COX1.

The phylogenetic analysis based on the mitogenomes of the 20 *Conus* species indicated that *C. marmoreus* and (*C. textile* and *C. gloriamaris*) were clustered together as a sister group (Figure 5). Our phylogenetic tree showed that *C. litteratus* and *C. quercinus* was a sister group to *C. virgo* (both PP = 1.00, BS = 99), and we considered the phylogenetic relation of *C. imperialis* and *C. tribblei* to be unsupported by our data, as it was given low node bootstrap values (PP = 0.79, BS = 50).

## 3. Discussion

Up to now, mitochondrial molecular markers, such as 16srRNA and COX1, have been widely used in the phylogenesis of metazoan [8,12]. However, the entire mitochondrial genome is currently the one that can provide systematic studies at the genome level molecular marker and be more informative than a single gene. The mitochondrion of *C. imperialis*, *C. litteratus*, *C. virgo*, and *C. marmoreus* in the South China Sea has not yet been reported. It is interesting to note that CR is difficult to amplify due to its complex structure, which prevented Taq polymerase for completing the PCR reactions in some species [16]. However, the primers designed in the trnF and cox3 genes at the boundaries of the CR in this study can be successfully amplified.

By comparison with the nucleotide composition of mitochondrial genomes of all available *Conus* species, the results indicated the entire AT content of the complete mitochondrion more than GC content. In addition, the tRNA^ser(S2)^ of the four newly sequenced mitogenomes lacked the DHU arm; however, the absence of the DHU arm in the second structure of tRNA^ser(S2)^ is commonly observed in mollusks [23,24,25]. In contrast to the majority of Watson–Crick base pairs, certain non-Watson–Crick base pairs (base pairs G-U) were present in the stems of the clover-leaf secondary structures; these mismatches are frequently found in the mitogenomes of other metazoan animals and can be corrected through editing processes [26,27]. In some studies, the characteristics of the mitochondrial genome, such as gene order, can be a useful marker for phylogenetic inference [28]. By comparison analysis, the characteristics of the mitochondrial genome, such as the locations of genes and the usage patterns of the initial and terminal codons, are conservative.

Three datasets (COX1, PCGs, and the complete mitogenome) were used to construct phylogenetic relationships to understand better the relationships of *Conus* species. To examine the impact of different methods and datasets on the topology of the tree and nodal support, independent phylogenetic studies were conducted. The result of DAMBE analysis showed that three datasets (COX1, PCGs, and the complete mitogenome) had a lower ISS value than the ISS.c (*p* ≤ 0.0001), which confirmed the suitability of phylogenetic analyses of these three datasets. Nodal supporting values for BI trees were consistently higher than those for ML trees for the same dataset, which is frequently the case in earlier research on many other taxa [16,17]. Among the phylogenetic trees constructed from the three datasets, the topology structure of the phylogenetic tree based on PCGs and the complete mitogenome was more stable than that based on COX1. In contrast to nodes based on PCGs and the complete mitogenome, nodes in the phylogenetic tree based on COX1 had low support values. The phylogenetic relations of *C. borgesi*, *C. tulipa*, and *C. textile* based on COX1 were inconsistent with previous viewpoints, but the phylogenetic relations of *C. borgesi*, *C. tulipa*, and *C. textile* based on PCGs and the complete mitogenome were consistent with previous viewpoints [16].

The phylogenetic analysis based on PCGs and the complete mitogenome of the 20 *Conus* species indicated that *C. guanche*, *C. infinitus*, *C. hybridus*, *C. unifasciatus*, and *C. borgesi* were a monophyletic group with high support (Figure 4), which is in consensus with previous viewpoints [16,17,29]. At the same time, ((*C. consors* and *C. striatus*) and *C. tulipa*) and (*C. betulinus* and (*C. marmoreus* and (*C. textile* and *C. gloriamaris*))) were clustered together as a sister group; such a pattern reconfirmed the conclusion presented by Juan E. Uribe et al. about *C. consors* and *C. textile* [16]. Therefore, our study supported that PCGs and the complete mitogenome are the two useful markers for phylogenetic inference of *Conus* species.

Due to the small number of *Conus* species used in this study, the result may be more conservative. Therefore, more species from the *Conus* species and more rapidly evolving molecular markers, including the mitogenome, are necessary to understand the phylogenetic relationship within the genus *Conus*.

## 4. Materials and Methods

### 4.1. Specimen Collection and DNA Extraction

Four different species of cone snails (*C. imperialis*, *C. litteratus*, *C. virgo*, and *C. marmoreus*) were collected in the South Sea of China. The mitochondrial genome of cone snails was extracted by an improved high-salt precipitation method. In brief, approximately 0.15 g of muscle tissue of the gastrofoot was weighed, and 1ml of Buffer I (2 mmol/L EDTA, 5 mmol/L Tris, 7 mmol/L sucrose, and 220 mmol/L dulcitol) was added to it, and the pH was adjusted to 7. Next, the tissue was homogenized in an ice-cold tissue grinder (ServiceBio, KZ-III-F, Wuhan, China) then centrifuged at 800× *g* for 10 min at 4 °C, and the supernatant was collected. This procedure was repeated twice, then the supernatant was centrifuged at 12,000× *g* for 10 min at 4 °C. The supernatant was discarded, and the precipitation was the isolated mitochondria. Then, 400 µL of Buffer Ⅱ (100 mmol/L Tris, 40 mmol/L Nacl, and 2 mmol/L EDTA), 16 µL of 20% SDS, and 16 µL of proteinase K (20 mg/mL) were added, after which the mitochondria was resuspended and incubated in a 60 °C water bath for at least 8 h until the solution was clear. Next, 300 of µL NaCl solution (40 mmol/L) was added and centrifuged at 8000× *g* for 10 min at 4 °C. The precipitation was discarded, and supernatant was collected. Then, isopropanol was added and centrifuged at 12,000× *g* for 15 min at 4 °C to precipitate the isolated mitochondrial DNA.

### 4.2. PCR Amplification and Sequencing

To obtain the full-length sequence of the cone snails’ mitochondrial genome DNA, five pairs of primers were designed by premier 5.0 software (Premier, Palo Alto, CA, USA) (Table S5). The target gene fragments were amplified by Polymerase Chain Reaction (PCR) using the mitochondrial genome as a template. The complete mitogenome was amplified by these five pairs of primers according to their positions on the mitochondrial genome, and there was an overlap between each pair of primers. Complete mitogenomes were amplified through a combination of standard and long PCRs. All PCR reactions were conducted in 50 μL of solution. The long PCR reactions contained 25 µL of Phanta Flash Master Mix (Vazyme, Nanjing, China), 2 µL of each primer (10 nmol/L), 2 µL of template DNA (100 ng/µL), and sterilized distilled water up to 50 µL. The following conditions were used to conduct long PCR: initial denaturing at 98 °C for 30 s, followed by 38 cycles of denaturing at 98 °C for 10 s, next annealing at 50 °C for 5 s and extending at 72 °C for 40 s, and then the final extension was held at 72 °C for 5 min. The standard PCR reactions contained 25 µL of Taq Master Mix (Vazyme, Nanjing, China), 2 µL of each primer (10 nmol/L), 2 µL of template DNA (100 ng/µL), and sterilized distilled water up to 50 µL. The following conditions were used to conduct standard PCR: initial denaturing step at 95 °C for 3 min, then 40 cycles of denaturing at 95 °C for 30 s, next annealing at 50 °C for 15 s and extending at 72 °C for 2 min, and a final extending step at 72 °C for 5 min. The amplified DNA was purified by agarose gel electrophoresis and isolated using Gel Extraction Kit (Omega Biotek, Norcross, GA, USA). Purified amplicons were cloned into PMD19-T vector (Takara, Kyoto, Japan), followed by being transferred into DH5α competent E. coli. The nucleotide sequence of the insert was sequenced using Sanger gene sequencing apparatus (model: ABI3730XL) by Tianyihuiyuan Biotech Company at Guangzhou in the province of Guangdong.

### 4.3. Genome Assembly and Annotation

The sequences corresponding to the different PCR amplified mt-genomes were trimmed, checked, and assembled by using Seqman 7.1.0 (DNASTAR, Inc., Madison, WI, USA) and DNAMAN V6 software (lynnon biosoft, San Ramon, CA, USA). The positions of the PCGs were discerned by using ORF Finder via NCBI (https://www.ncbi.nlm.nih.gov/orffinder/ (accessed on 7 May 2023)), while the invertebrate mitochondrial genetic code was selected and confirmed by the MITOS web server. The MITOS web server [30] and tRNAscan-SE Online Search Server [31] were used to identify the tRNAs and their secondary structures, and then the results were confirmed by manual proofreading. Additionally, the locations of rRNAs and the (control region) CR were determined according to the boundaries of tRNAs and homology comparison. The complete mitochondrion was further verified by the homology comparison [15,32,33,34,35,36]. The map of the mitochondrial genome was visualized and edited by the CGView [37] (https://proksee.ca/ (accessed on 7 May 2023)).

Strand compositional asymmetry was analyzed by calculating AT and GC skews using formulas: AT-skew = (A − T)/(A + T) and GC-skew = (G − C)/(G + C) [38]. MEGA 6 was used to analyze the relative synonymous codon usage (RSCU) and nucleotide substitution statistics [39].

### 4.4. Phylogenetic Analysis

In order to explore their phylogenetic relationships, the newly sequenced mitochondrial genomes of 4 species from cone snails in this study and another 16 complete mitochondrial genomes of cone snails were included in the phylogenetic analysis (Table S4), and their complete mitogenomes were attained from the GenBank database (https://www.ncbi.nlm.nih.gov/genbank/ (accessed on 7 May 2023)). The multiple sequence alignments of COX1, 13 PCGs, and the complete mitogenome were conducted by Mafft in PhyloSuite. The individual alignments were then concatenated, followed by the elimination of the poorly aligned and highly divergent regions by PhyloSuite [40]. The index of substitution saturation tests was calculated by DAMBE 5 [41].

Three datasets were constructed: 13 PCGs, COX1, and the complete mitogenome. For each dataset, Bayesian Inference (BI) and Maximum Likelihood (ML) techniques were constructed to determine whether the datasets were susceptible to the interference methods. For the ML analyses, partition-specific models and the best-fit partitioning scheme were analyzed by ModelFinder in Phylosuite. Standard bootstrapping of 1000 replicates was used to evaluate the clade support. For the BI analyses, partition-specific models and the best-fit partitioning scheme analyzed by ModelFinder in Phylosuite were utilized. By running 4 Markov Chain Monte Carlo (MCMC) chains of 1 million generations twice, sampling every 98 generations, and tossing out the first 26% of the generations as burn-in, the posterior probabilities (PP) were determined in a consensus tree. The iTOL was used to modify and visualize the phylogenetic trees [42].

## 5. Conclusions

In this study, a comparative analysis of mitogenomes and a phylogenetic analysis were carried out among the mitogenomes of these four *Conus* species. The results showed that the general genomic features found in these four *Conus* species are similar to the previous studies on *Conus*. In addition, unconventional base pairs of *C. litteratus*, *C. imperialis*, *C. marmoreus*, and *C. virgo* were found in the secondary structures of tRNAs, which will be corrected in the subsequent editing stage. Almost all the tRNAs of our analysis could be folded into a typical clover-leaf secondary structure, except for tRNAser(S2), which lacked the DHU arm. We studied and analyzed the genetic relationships of four species of cone snails (*C. litteratus*, *C. imperialis*, *C. marmoreus*, and *C. virgo*) in the South China Sea. The phylogenetic analysis based on COX1, PCGs, and the complete mitogenome clearly showed that *C. Guanche*, *C. infinitus*, *C. hybridus*, *C. unifasciatus*, and *C. borgesi* were a monophyletic group, and (*C. litteratus* and *C. quercinus*) and *C. virgo* were clustered together as a sister group (PP = 1, BS = 99), which was supported by our result. Our data did not support the relationship of *C. imperialis* and *C. tribblei*, given the low node bootstrap values of *C. imperialis* and *C. tribblei* (PP = 0.79, BS = 50). By comparison, our study supported that PCGs and complete mitogenome are the two useful markers for phylogenetic inference of *Conus* species.

## Figures and Tables

**Table 1:** Nucleotide composition of the mitogenomes for 20 species of *Conus*.

Species	A + T%	AT-Skew	GC-Skew	Species	A + T%	AT-Skew	GC-Skew
C. virgo *	64.42	−0.17	0.15	*C. unifasciatus*	66.08	−0.16	0.11
C. imperialis *	65.96	−0.15	0.1	*C. borgesi*	67.15	−0.15	0.11
C. litteratus *	66.05	−0.16	0.12	*C. quercinus*	66.47	−0.15	0.12
C. marmoreus *	66.81	−0.15	0.12	*C. striatus*	64.54	−0.2	0.17
*C. consors*	67.12	−0.17	0.18	*C. tribblei*	65.95	−0.15	0.1
*C. gloriamaris*	66.27	−0.16	0.11	*C. tulipa*	66.4	−0.14	0.1
*C. textile*	65.21	−0.16	0.1	*C. ebraeus*	66.55	−0.12	0.06
*C. guanche*	66.07	−0.16	0.12	*C. betulinus*	63.93	−0.2	0.19
*C. infinitus*	67.19	−0.15	0.12	*C. capitaneus*	62.23	−0.18	0.14
*C. hybridus*	66.04	−0.16	0.11	*C. miles*	61.84	−0.17	0.14

* represents mitogenome sequences that were obtained in this study.

## Data Availability

Four new mitogenome data in this study have been submitted to the GenBank database under accession numbers as follows: OR033159 for mitogenome of *Conus virgo*, OR033160 for mitogenome of *Conus litteratus*, and OR033161 for mitogenome of *Conus imperialis*, OR033162 for mitogenome of *Conus marmoreus*.

## Supplementary Materials

The following supporting information can be downloaded at: https://www.mdpi.com/article/10.3390/ijms24119411/s1.
