# Supplementary material for: Mitogenome Characterization of Four Conus Species and Comparative Analysis
Source: Int J Mol Sci. 2023 May 28;24(11):9411. doi: 10.3390/ijms24119411 (PMC10253580; doi:10.3390/ijms24119411)
Supplement: Supplementary file 1 [file ijms-24-09411-s001.zip › ijms-2413745-Supplementary Material.pdf]

International Journal of *Molecular Sciences*

Article

Mitogenome Characterization of Four *Conus* Species and Comparative Analysis

Hao Wang[1], Xiaopeng Zhu[2], Yuepeng Liu[1], Sulan Luo[1,2,*] and Dongting Zhangsun[1,2,*]

Table S1. Annotation of the mitochondrial genomes of *C. litteratus*, *C. imperialis*, *C. marmoreus*, *C. virgo*.

Feature	Strand	Anticode	*C. imperialis*		*C. litteratus*		*C. marmoreus*		*C. virgo*	
			Position	In	Position	In	Position	In	Position	In
cox1	J	-	1-1548	126	1-1548	155	1-1548	157	1-1548	144
cox2	J	-	1675-2361	0	1704-2390	0	1706-2392	0	1693-2379	0
trnD (gtc)	J	GTC	2362-2428	0	2391-2457	0	2393-2458	1	2380-2445	0
atp8	J	-	2429-2590	7	2458-2619	6	2460-2621	6	2446-2607	9
atp6	J	-	2598-3293	35	2626-3321	36	2628-3323	35	2617-3312	35
trnM (cat)	N	CAT	3329-3395	9	3358-3425	6	3359-3426	9	3348-3414	9
trnY (gta)	N	GTA	3405-3472	0	3432-3497	1	3436-3501	2	3424-3489	2
trnC (gca)	N	GCA	3473-3537	0	3499-3564	0	3504-3567	0	3492-3556	1
trnW (tca)	N	TCA	3538-3603	-2	3565-3630	-2	3568-3633	1	3558-3623	1
trnQ (ttg)	N	TTG	3602-3667	18	3629-3693	13	3635-3692	15	3625-3682	19
trnG (tcc)	N	TCC	3686-3751	1	3707-3772	1	3708-3773	1	3702-3767	1
trnE (ttc)	N	TTC	3753-3819	0	3774-3840	0	3775-3842	0	3769-3833	0
rrnS	J	-	3820-4769	0	3841-4793	0	3843-4798	0	3834-4785	0
trnV (tac)	J	TAC	4770-4836	0	4794-4860	0	4799-4865	0	4786-4852	0
rrnL	J	-	4837-6201	0	4861-6226	0	4866-6234	0	4853-6228	0
trnL1 (tag)	J	TAG	6202-6271	8	6227-6296	5	6235-6304	6	6229-6298	6
trnL2 (taa)	J	TAA	6280-6348	0	6302-6370	0	6311-6379	0	6311-6379	0
nad1	J	-	6349-7290	3	6371-7312	4	6380-7321	4	6380-7321	4
trnP (tgg)	J	TGG	7294-7359	0	7317-7383	0	7326-7392	0	7326-7392	0
nad6	J	-	7360-7860	14	7384-7884	12	7393-7893	11	7393-7893	11
cob	J	-	7875-9014	13	7897-9036	12	7905-9044	11	7905-9044	11

trnS2 (tga)	J	TGA	9028-9092	10	9049-9113	11	9056-9120	11	9056-9120	10
trnT (tgt)	N	TGT	9103-9170	21	9125-9195	13	9132-9198	18	9131-9197	21
nad4l	J	-	9192-9488	-7	9209-9505	-7	9217-9513	-7	9219-9515	-7
nad4	J	-	9482-10864	-1	9499-10881	-1	9507-10889	-1	9509-10891	0
trnH (gtg)	J	GTG	10864-10930	0	10881-10947	1	10889-10955	0	10892-10958	0
nad5	J	-	10931-12646	-1	10949-12661	2	10956-12671	-1	10959-12674	-1
trnF(gaa)	J	GAA	12646-12710	120	12664-12729	128	12671-12735	140	12674-12738	140
cox3	J	-	12831-13610	20	12858-13637	30	12876-13655	27	12879-13658	29
trnK (ttt)	J	TTT	13631-13699	5	13668-13738	5	13683-13753	19	13688-13758	5
trnA (tgc)	J	TGC	13705-13771	13	13744-13810	36	13773-13839	17	13764-13830	22
trnR (tcg)	J	TCG	13785-13853	9	13847-13915	13	13857-13925	13	13853-13921	16
trnN (gtt)	J	GTT	13863-13931	9	13929-13997	6	13939-14007	9	13938-14007	18
trnI (gat)	J	GAT	13941-14010	4	14004-14073	2	14017-14087	5	14026-14095	5
nad3	J	-	14015-14368	9	14076-14429	17	14093-14446	10	14101-14454	17
trnS1 (gct)	J	GCT	14378-14445	0	14447-14514	0	14457-14524	0	14472-14539	0
nad2	J	-	14446-15501	4	14515-15569,1	-1	14525-15579,1	-1	14540-15594,1	-1

Note: The arrangement of the genes in the table corresponds to the arrangement of the genes in the mitochondrion. The position number refers to the position on the majority. J and N refer to the majority strand and the minority strand.

Table. S2. Codon usages of the protein-coding genes in the mitogenomes of 20 *Conus* species

	**cox1**	**cox2**	**atp8**	**atp6**	**nad1**	**nad6**	**cob**
	start/stop codon	start/stop codon	start/stop codon	start/stop codon	start/stop codon	start/stop codon	start/stop codon
C. betulinus	ATG/TAG	ATG/T	ATG/TAA	ATG/TAA	ATG/TAA	ATG/TAG	ATG/TAG
C. capitaneus	ATG/TAG	ATG/TAG	ATG/TAA	ATG/TAG	ATG/TAA	ATG/TAA	ATG/TAG
C. consors	ATG/TAA	ATG/TAA	ATG/TAA	ATG/TAA	ATG/TAA	ATG/TAA	ATG/TAA
C. textile	ATG/TAG	ATG/TAA	ATG/TAG	ATG/TAG	ATG/TAG	ATG/TAG	ATG/TAA
C. gloriamaris	ATG/TAA	ATG/TAG	ATG/TAG	ATG/TAG	ATG/TAG	ATG/TAG	ATG/TAA
C. borgesi	ATG/TAA	ATG/TAG	ATG/TAG	ATG/TAA	ATG/TAA	ATG/TAA	ATG/TAG
C. guanche	ATG/TAA	ATG/TAG	ATG/TAG	ATG/TAG	ATG/TAA	ATG/TAA	ATG/TAG
C. infinitus	ATG/TAA	ATG/TAA	ATG/TAG	ATG/TAA	ATG/TAA	ATG/TAA	ATG/TAA
C. hybridus	ATG/TAA	ATG/TAG	ATG/TAA	ATG/TAG	ATG/TAA	ATG/TAA	ATG/TAG
C. quercinus	ATG/TAG	ATG/TAA	ATG/TAA	ATG/TAA	ATG/TAG	ATG/TAA	ATG/TAA
C. striatus	ATG/TAG	ATG/TAA	ATG/TAG	ATG/TAA	ATG/TAG	ATG/TAA	ATG/TAA
C. tribblei	ATG/TAA	ATG/TAG	ATG/TAG	ATG/TAG	ATG/TAA	ATG/TAA	ATG/TAA
C. tulipa	ATG/TAA	ATG/TAA	ATG/TAA	ATG/TAA	ATG/TAG	ATG/TAA	ATG/TAA
C. unifasciatus	ATG/TAA	ATG/TAG	ATG/TAG	ATG/TAG	ATG/TAA	ATG/TAA	ATG/TAG
C. miles	ATG/TAG	ATG/TAG	ATG/TAA	ATG/TAA	ATG/TAA	ATG/TAA	ATG/TAG
C. edraeus	ATG/TAA	ATG/TAG	ATG/TAA	ATG/TAG	ATG/TAA	ATG/TAG	ATG/TAG
*C. imperialis**	ATG/TAA	ATG/TAA	ATG/TAG	ATG/TAG	ATG/TAG	ATG/TAA	ATG/TAG
*C. litteratus**	ATG/TAA	ATG/TAG	ATG/TAA	ATG/TAG	ATG/TAA	ATG/TAA	ATG/TAG
*C. marmoreus**	ATG/TAA	ATG/TAG	ATG/TAA	ATG/TAG	ATG/TAA	ATG/TAA	ATG/TAA

*C. virgo**	ATG/TAG	ATG/TAA	ATG/TAG	ATG/TAA	ATG/TAG	ATG/TAG	ATG/TAA

Note: the red refers incomplete terminal codons. "*" refers to the newly sequenced mitogenome *Conus* species

Table. S2. (continued)

	nad4l	nad4	nad5	cox3	nad3	nad2
	start/stop codon	start/stop codon	start/stop codon	start/stop codon	start/stop codon	start/stop codon
C. betulinus	ATG/TAG	GTG/TA	ATG/TAA	ATG/TAA	ATG/TAG	ATG/TAA
C. capitaneus	ATG/TAG	GTG/TAA	ATG/TAG	ATG/TAA	ATG/TAG	ATG/TAA
C. consors	ATG/TAG	ATG/TAG	ATG/TAA	ATG/TAA	ATG/TAA	ATG/TAA
C. textile	ATG/TAG	ATG/TAG	ATG/TAA	ATG/TAA	ATG/TAG	ATG/TAA
C. gloriamaris	ATG/TAG	ATG/TAG	ATG/TAA	ATG/TAA	ATG/TAG	ATG/TAA
C. borgesi	ATG/TAG	ATA/TAG	ATG/TAA	ATG/TAG	ATG/TAA	ATG/TAA
C. guanche	ATG/TAG	ATG/TA	ATG/TAA	ATG/TAA	ATG/TAA	ATG/TAA
C. infinitus	ATG/TAG	ATG/TA	ATG/TAA	ATG/TAA	ATG/TAA	ATG/TAA
C. hybridus	ATG/TAG	ATG/TA	ATG/TAA	ATG/TAA	ATG/TAA	ATG/TAA
C. quercinus	ATG/TAG	ATG/TAG	ATG/TAA	ATG/TAA	ATG/TAG	ATG/TAA
C. striatus	ATG/TAG	GTG/TAG	ATG/TAA	ATG/TAG	ATG/TAA	ATG/TAA
C. tribblei	ATG/TAG	ATG/TAG	ATG/TAA	ATG/TAG	ATG/TAA	ATG/TAA
C. tulipa	ATG/TAG	ATG/TAG	ATG/TAA	ATG/TAG	ATG/TAA	ATG/TAA
C. unifasciatus	ATG/TAG	ATG/TA	ATG/TAA	ATG/TAA	ATG/TAA	ATG/TAA
C. miles	ATG/TAG	ATG/TAA	ATG/TAG	ATG/TAA	ATG/TAG	ATG /TAA
C. edraeus	ATG/TAG	ATG/TAG	ATG/TAA	ATG/TAA	ATG/TAG	ATG /TA
*C. imperialis**	ATG/TAG	GTG/TAG	ATG/TAA	ATG/TAA	ATG/TAA	ATG/TAA
*C. litteratus**	ATG/TAG	ATG/TAG	ATG/TAA	ATG/TAA	ATG/TAG	ATG/TAA
*C. marmoreus**	ATG/TAG	ATG/TAG	ATG/TAA	ATG/TAG	ATG/TAA	ATG/TAA
*C. virgo**	ATG/TAG	ATG/TAA	ATG/TAA	ATG/TAA	ATG/TAG	ATG/TAA

Note: the red refers incomplete terminal codons. “*” refers to the newly sequenced mitogenome *Conus* species

Table. S3. The result of nucleotide saturation of different datasets

dataset	Iss	Iss.c	P
PCGs	0.2279	0.8483	0.0000
COX1	0.1764	0.7852	0.0000
Complete mitogenome	0.2173	0.8540	0.0000

Table. S4. Complete MTDNA sequence data for phylogenetic analysis

Species	Length (bp)	GenBank Acc. No.[a]	Species	Length (bp)	GenBank Acc. No.[a]
C. imperialis	15505	This work	*C. textile*	15756	KX155574.1
C. litteratus	15569	This work	*C. guanche*	15506	KY801847.1
C. virgo	15594	This work	*C. infinitus*	15522	KY864967.1
C. marmoreus	15579	This work	*C. hybridus*	15507	KY801863.1
C. capitaneus	15829	NC_030354.1(outgroup)	*C. unifasciatus*	15506	KY801860.1
C. miles	16243	NC_063770.1(outgroup)	*C. borgesi*	15536	EU827198.1
C. betulinus	16240	NC_039922.1	*C. quercinus*	16430	NC_035007.1
C. consors	16112	NC_023460.1	*C. striatus*	15738	KX156937.1
C. gloriamaris	15774	NC_030213.1	*C. tribblei*	15570	NC_027957.1

[a]GenBank Acc. No. refers to gene bank accession of NCBI.

Table. S5. Five pairs of primers designed in this study

Primer	Sequence5’-3’
11F	TCTATTGGKCATTTAGGDTG
11R	ATACHTRAAATCAGAGCCAAGT
52F	TYAGGATGAAAACCTAAYGTGC
52R	CCDCCTCATACYCAYTCTACYA
63F	GCTTTTAGRTCTGTAATTCATAT
63R	AGTTGTTACCCTAGCATCTT
43F	AGAGCRTRGCRCTGAAGATGC
43R	ATTCCHGCAGGAGGYCARCAHGAHCC
34F	TCCRTTTCATTTRGTTGARTTTAG
34R	GTAGAAAATARCCATCGCAT

Table S6. The partition and best-fit partition models used in this study

Dataset	Partition and best-fit partition model
COX1-BI	**GTR+F+G4**
COX1-ML	**TIM3+F+I+G4**
PCGs-BI	**GTR+F+I+G4** (atp6_codon1, atp8_codon1, atp8_codon2, nad1_codon1, nad2_codon1, nad3_codon1, nad4L_codon1, nad4_codon1, nad5_codon1, nad6_codon1)<br>**GTR+F+I+G4** (atp6_codon2, cox1_codon2, cox2_codon2, cox3_codon2, nad1_codon2, nad4L_codon2)<br>**HKY+F+I+G4** (atp6_codon3, cytb_codon3, nad6_codon3,)<br>**HKY+F+I+G4** (atp8_codon3, cox2_codon3, nad4L_codon3)<br>**GTR+F+I+G4** (cox1_codon1, cox2_codon1, cox3_codon1,)<br>**HKY+F+I+G4** (cox1_codon3, cox3_codon3, nad1_codon3, nad2_codon3, nad3_codon3)<br>**SYM+G4** (cytb_codon1)<br>**F81+F+G4** (cytb_codon2)<br>**GTR+F+I+G4** (nad2_codon2, nad3_codon2, nad4_codon2, nad5_codon2, nad6_codon2)<br>**GTR+F+I+G4** (nad4_codon3, nad5_codon3)
PCGs-ML	**GTR+F+I+G4** (atp6, nad4L, nad4, nad5, nad6)<br>**TIM3+F+I+G4** (atp8, nad2)<br>**TIM+F+I+G4** (cox1, cox2, cox3, nad1, nad3)<br>**HKY+F+G4** (cytb)
Complete mitogenome-BI	**GTR+F+I+G4**
Complete mitogenome-ML	**GTR+F+I+G4**

Note: the bold word refers to the best-fit partition model. The content in "()" refers to the partition.

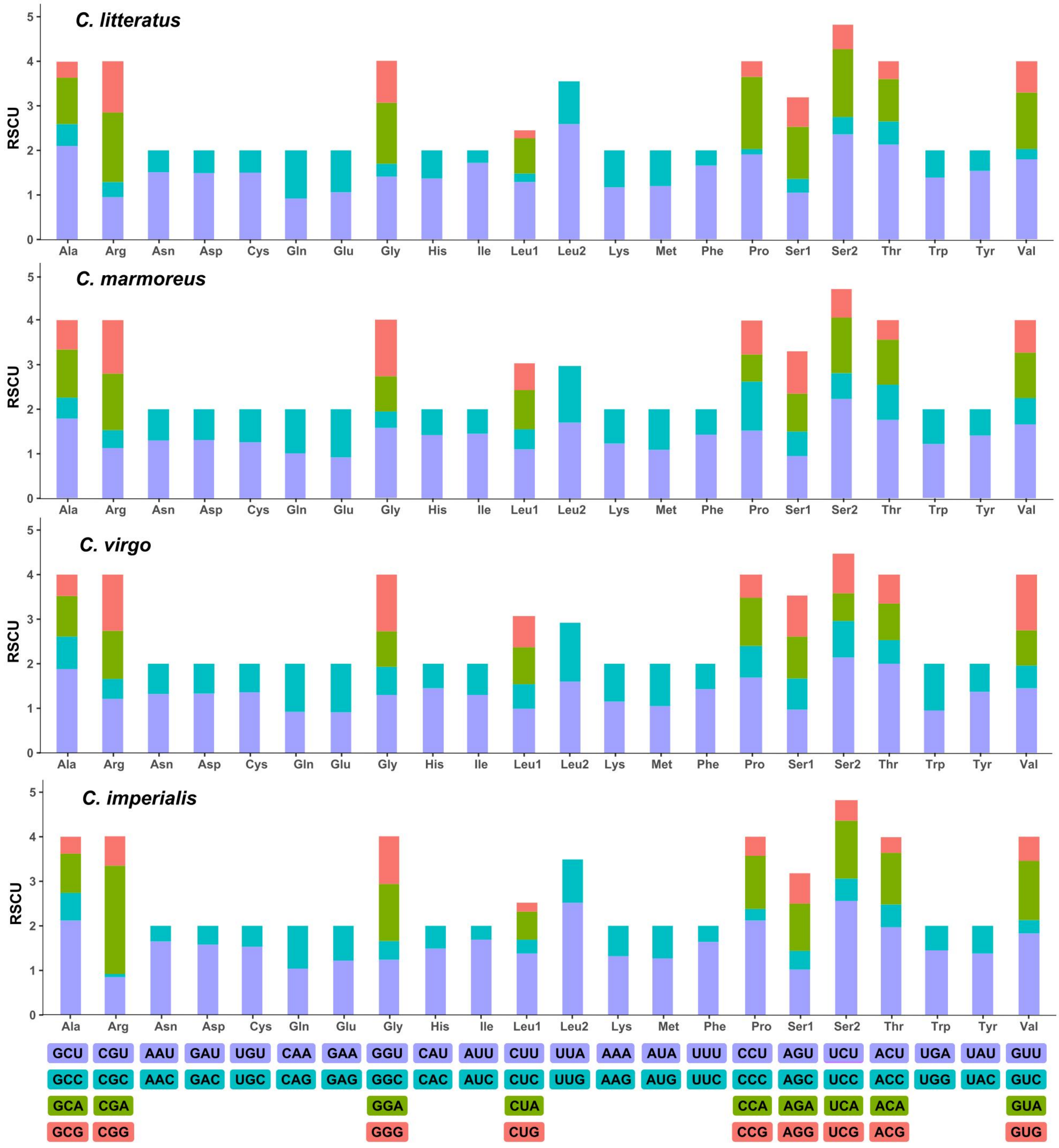

Fig. S1. The relative synonymous codon usage (RSCU) of the four newly sequenced mitogenomes. The order of amino acids below the X-axis and the color of codons correspond to their order of the letters.

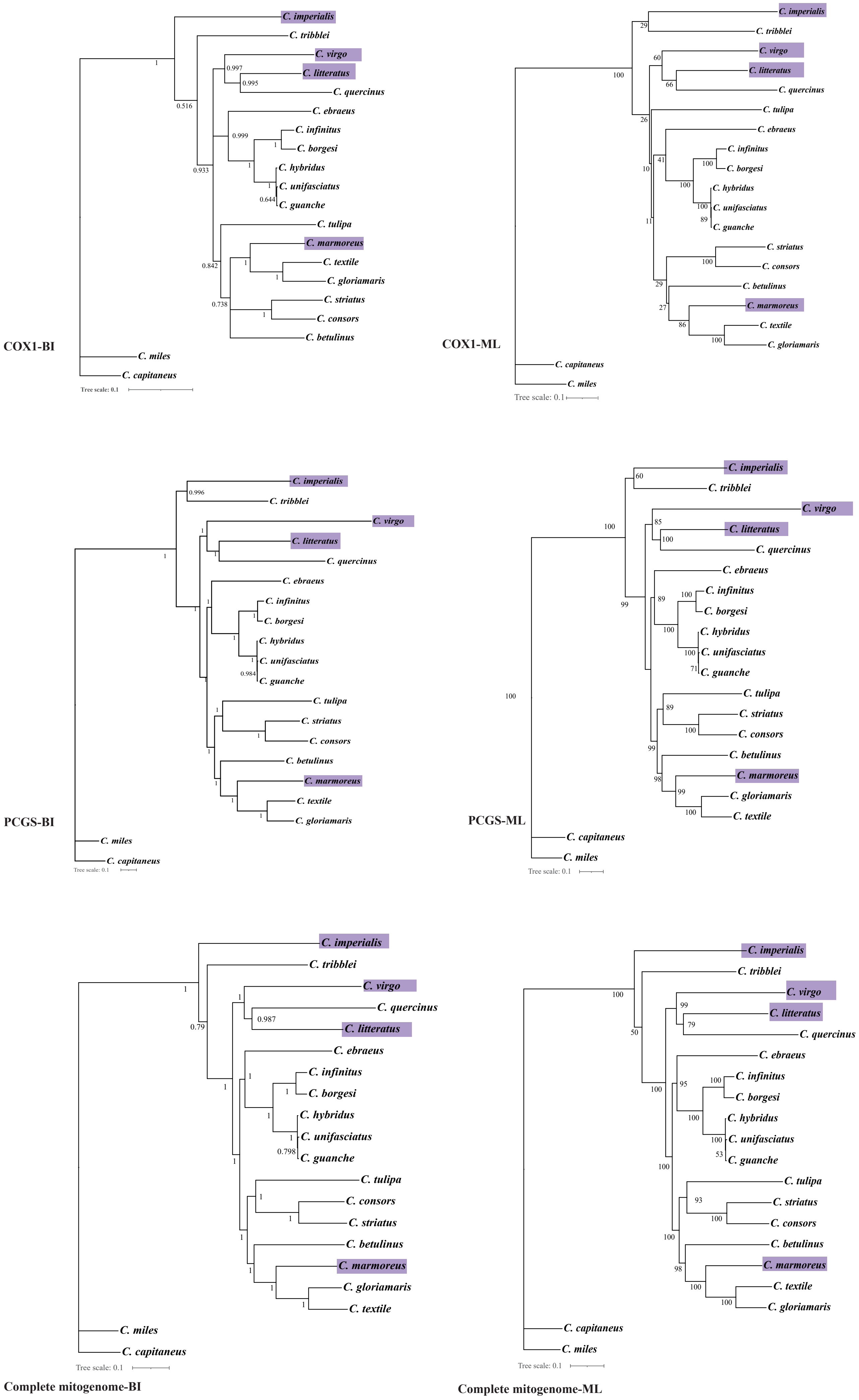
COX1-BI
C. imperialis
C. tribblei
C. virgo
C. litteratus
C. quercinus
C. ebraeus
C. infinitus
C. borgesi
C. hybridus
C. unifasciatus
C. guanche
C. tulipa
C. marmoreus
C. textile
C. gloriamaris
C. striatus
C. consors
C. betulinus
C. miles
C. capitaneus
Tree scale: 0.1
COX1-ML
C. imperialis
C. tribblei
C. virgo
C. litteratus
C. quercinus
C. tulipa
C. ebraeus
C. infinitus
C. borgesi
C. hybridus
C. unifasciatus
C. guanche
C. striatus
C. consors
C. betulinus
C. marmoreus
C. textile
C. gloriamaris
C. capitaneus
C. miles
Tree scale: 0.1
PCGS-BI
C. imperialis
C. tribblei
C. virgo
C. litteratus
C. quercinus
C. ebraeus
C. infinitus
C. borgesi
C. hybridus
C. unifasciatus
C. guanche
C. tulipa
C. striatus
C. consors
C. betulinus
C. marmoreus
C. textile
C. gloriamaris
C. miles
C. capitaneus
Tree scale: 0.1
PCGS-ML
C. imperialis
C. tribblei
C. virgo
C. litteratus
C. quercinus
C. ebraeus
C. infinitus
C. borgesi
C. hybridus
C. unifasciatus
C. guanche
C. tulipa
C. striatus
C. consors
C. betulinus
C. marmoreus
C. gloriamaris
C. textile
C. capitaneus
C. miles
Tree scale: 0.1
C. imperialis
C. tribblei
C. virgo
C. quercinus
C. litteratus
C. ebraeus
C. infinitus
C. borgesi
C. hybridus
C. unifasciatus
C. guanche
C. tulipa
C. consors
C. striatus
C. betulinus
C. marmoreus
C. gloriamaris
C. textile
C. miles
C. capitaneus
Tree scale: 0.1
Complete mitogenome-BI
C. imperialis
C. tribblei
C. virgo
C. litteratus
C. quercinus
C. ebraeus
C. infinitus
C. borgesi
C. hybridus
C. unifasciatus
C. guanche
C. tulipa
C. striatus
C. consors
C. betulinus
C. marmoreus
C. textile
C. gloriamaris
C. capitaneus
C. miles
Tree scale: 0.1
Complete mitogenome-ML

Fig. S2. Phylogenetic tree constructed by different datasets and analytical approaches. Tree topologies constructed by the three methods are in almost complete agreement. ML bootstrap value (BV) and BI posterior probability (PP) are separated by a slash on node. The reconstructed ML/BI phylogram using *C. capitaneus*, *C. miles* as outgroup. The purple refers to the species whose mitogenome were newly sequenced in this work.
